# A Scoping Review Protocol to Map Knowledge and Attitudes about Patient Safety among Students in Healthcare Undergraduate Courses

**DOI:** 10.3390/nursrep14030150

**Published:** 2024-08-20

**Authors:** Fabricio Renato Teixeira Valença, Martins Fideles dos Santos Neto, Yuri Sacardo, Denise Cristina Móz Vaz Oliani, Emerson Roberto dos Santos, Vânia Maria Sabadoto Brienze, Alba Regina de Abreu Lima, Júlio César André, Vania Del Arco Paschoal

**Affiliations:** 1 Center for Studies and Development of Health Education, Faculty of Medicine of São José do Rio Preto (CEDES/FAMERP), São José do Rio Preto 15090-000, Brazil; emerson.santos@edu.famerp.br (E.R.d.S.); vania.brienze@hospitaldebase.com.br (V.M.S.B.); alba.lima09@gmail.com (A.R.d.A.L.); julio.andre@edu.famerp.br (J.C.A.); 2 Barretos Cancer Hospital, Pio XII Foundation, HCB, Barretos 14784-370, Brazil; martins_neto17@hotmail.com; 3 Faculty of Medicine of São José do Rio Preto, São José do Rio Preto 15090-000, Brazil; enfyurisacardo@gmail.com (Y.S.); vaz.oliani@gmail.com (D.C.M.V.O.); vania@famerp.br (V.D.A.P.); 4 University Hospital Center Cova da Beira, University of Beira Interior, 6200-251 Covilhã, Portugal

**Keywords:** nursing, patient safety, students, education, knowledge, attitude, scoping review protocol

## Abstract

Patient safety is a global challenge in healthcare, with adverse events representing a significant concern. The integration of patient safety education in undergraduate curricula is crucial in developing a culture of safety and safe practices among future professionals. However, there is a gap in research assessing the levels of knowledge and attitudes about patient safety among undergraduate students in healthcare using validated instruments. This scoping review aims to map the levels of knowledge and attitudes about patient safety among students in healthcare courses, allowing for national and international comparisons. The review will follow the methodological frameworks proposed by the Joanna Briggs Institute (JBI) for scoping reviews. The search will include published and unpublished studies in six databases, with no date or language restrictions. This scoping review was prospectively registered with the Open Science Framework on 17 July 2024. This scoping review will provide a comprehensive overview of knowledge and attitudes about patient safety among undergraduate students in healthcare, identifying gaps in patient safety education and areas for improvement in curricula. The results may guide teachers in creating teaching strategies to prepare future healthcare professionals, reducing knowledge gaps and improving the quality of care.

## 1. Introduction

Patient safety is a global challenge for healthcare systems, with inherent risks in every action of the care process [1]. Adverse events, defined as unintentional harm resulting from healthcare, represent a significant concern [2]. In recent decades, scholars have contributed to advances in the quality of care and patient safety [3]. The World Health Organization (WHO) defines patient safety as a set of activities aimed at reducing risks and preventable harm in healthcare [1].

To achieve positive outcomes in care, the involvement of all professionals in the domain of patient safety is essential, as well as the improvement of training in this area by educational institutions [4]. The “Global Patient Safety Action Plan 2021–2030”, adopted by the WHO, reinforces patient safety as an essential component in healthcare systems [1,5].

Reflective, critical, and participatory teaching is widely agreed among health educators to promote competencies, attitudes, and skills [6]. Strategies to improve the quality of care and minimize risks include the implementation of patient safety in undergraduate curricula [7,8]. International organizations recommend teaching patient safety during higher education in health [9], as an important strategy strengthen the culture of safety and develop safe practices professionals [10].

In 2011, the WHO launched the multi-professional patient safety curriculum guide, providing educational approaches and teaching and assessment methods for integration into undergraduate health curricula [11]. The assessment of students’ self-perceived competence is crucial to identify gaps in patient safety training and assist in adapting curricula [12].

A preliminary search did not identify systematic or scoping reviews on the levels of knowledge and attitudes towards patient safety among undergraduate students in healthcare. There is a gap in research assessing these aspects using validated instruments [13,14,15].

This scoping review aims to map the levels of knowledge and attitudes about patient safety among students in healthcare courses, allowing for national and international comparisons. By providing a comprehensive overview of the measurement of these aspects and clarifying key concepts, this review will reveal research gaps in this topic.

This scoping study aims to examine and catalog the understanding and perspectives of undergraduate healthcare students regarding patient safety. Our objective is to delineate the current landscape of knowledge and attitudes of these future professionals on this crucial topic, providing valuable insights for healthcare educators and policy makers. The research question is “What are the levels of knowledge and attitudes about patient safety among undergraduate students in healthcare?”.

## 2. Methods

This review will follow the methodological frameworks proposed by the Joanna Briggs Institute (JBI) [16,17,18] for scoping reviews. The review process will involve (1) identifying the research question; (2) identifying relevant studies; (3) study selection; (4) data extraction; (5) synthesizing and reporting the results. The review will follow the PRISMA-ScR (PRISMA Extension for Scoping Reviews) recommendations, and the protocol was prospectively registered in the Open Science Framework (OSF) [19].

### 2.1. Inclusion Criteria

#### 2.1.1. Participants

Studies with undergraduate students from any healthcare course, year, institution, and country will be considered.

#### 2.1.2. Concept

The concept of interest is knowledge and attitudes about patient safety, attributes that healthcare professionals must develop to act preventively and promote a positive culture of safety. Results assessing the levels of these aspects will be included, regardless of the methodological approach (quantitative or qualitative) used in the studies.

#### 2.1.3. Context

The context is students from any healthcare course, country, and type of institution (public or private).

### 2.2. Types of Sources

Primary quantitative and qualitative studies, mixed-methods studies, and gray literature will be considered. Studies outside the scope, duplicate publications, letters, editorials, and opinion articles will be excluded.

### 2.3. Search Strategy

The search will include published and unpublished studies in the following databases: BVS, Cochrane Library, EMBASE, PubMed, Scopus, and Web of Science. Additionally, the search will include the following review databases: the Open Science Framework (OSF) and the International Platform of Registered Systematic Review and Meta-Analysis Protocols (INPLASY). Descriptors in English, Portuguese, and Spanish will be used, combined with Boolean operators (Table 1). There will be no date or language restrictions.

The search strategy will be developed using the following steps:

1. An initial limited search of PubMed and CINAHL will be undertaken to identify articles on the topic;
2. The text words contained in the titles and abstracts of relevant articles and the index terms used to describe the articles will be used to develop a full search strategy;
3. The search strategy, including all identified keywords and index terms, will be adapted for each included information source;
4. The reference lists of all included sources of evidence will be screened for additional studies.

The full search strategy for PubMed is detailed below.

The full search strategies for all databases will be provided in Appendix A of the final review.

### 2.4. Study Selection

The selection will be performed by two independent reviewers using the Rayyan application (software as a service—SaaS or a web application—that can be accessed at https://www.rayyan.ai/) [20]. After screening titles and abstracts, potentially relevant studies will be read in full. Discrepancies will be resolved by consensus or a third reviewer. The process will follow PRISMA [21] and will be presented in a flowchart.

### 2.5. Data Extraction

Data will be extracted using a standardized instrument developed for this review [22], including information on the authorship, year, country, objective, design, population, investigation details, outcomes, results, and conclusions (Table 2).

### 2.6. Data Analysis and Presentation

The data synthesis will be narrative, with tables and graphs used to summarize the findings [23]. Qualitative analyses, such as thematic analysis, may identify recurring themes [23]. If possible, quantitative analyses, such as meta-analyses, will be considered to combine results from similar studies [24].

## 3. Discussion

This scoping review will provide a comprehensive overview of the knowledge and attitudes about patient safety among undergraduate students in healthcare. Expected results include the identification of gaps in patient safety education and areas for improvement in curricula. Potential limitations may include the heterogeneity of studies and the scarcity of research with the target population.

## 4. Conclusions

This scoping review is important in mapping the levels of knowledge and attitudes about patient safety among students, providing suggestions to improve education on this topic. The results may guide teachers in creating teaching strategies to prepare future healthcare professionals, reducing knowledge gaps and improving the quality of care.

## Figures and Tables

**Table 1:** Search strategy for PubMed.

Database	Search Strategy
PubMed	(“Patient Safety”[Title/Abstract] OR “Patient Safeties”[Title/Abstract] OR “Safeties, Patient”[Title/Abstract] OR “Safety, Patient”[Title/Abstract]) AND (Students[Title/Abstract] OR Student[Title/Abstract] OR “School Enrollment”[Title/Abstract] OR “Enrollment, School”[Title/Abstract] OR “Enrollments, School”[Title/Abstract] OR “School Enrollments”[Title/Abstract] OR Learners[Title/Abstract] OR Scholars[Title/Abstract] OR Undergraduates[Title/Abstract] OR Graduates[Title/Abstract] OR Academics[Title/Abstract] OR Interns[Title/Abstract] OR Apprentices[Title/Abstract]) AND (Instrumentation[Title/Abstract] OR Instruments[Title/Abstract] OR Implements[Title/Abstract] OR Devices[Title/Abstract] OR Apparatus[Title/Abstract] OR Equipment[Title/Abstract] OR Utensils[Title/Abstract] OR Gadgets[Title/Abstract] OR Appliances[Title/Abstract] OR Contrivances[Title/Abstract] OR Mechanisms[Title/Abstract] OR Machines[Title/Abstract] OR Apparatuses[Title/Abstract] OR Gear[Title/Abstract] OR Hardware[Title/Abstract] OR Resources[Title/Abstract])

Source: Author.

**Table 2:** Data extraction instrument.

**Study Identification**						
Journal/impact factor	Title	Author	Year	Country	Language	Objective
**Research Participants**						
Course/period/institution	Sample size	Analysis method (scoring/classification system)			Inclusion criteria	Exclusion criteria

Source: Author.

## Data Availability

The data presented in this study are all available in this manuscript.

## Public Involvement Statement

No public involvement in any aspect of this research.

## Guidelines and Standards Statement

This manuscript was drafted against the Preferred Reporting Items for Systematic Review and Meta-Analysis (PRISMA) extension for protocol (PRISMA-P).

## Appendix A

**Table A1:** Search strategy for the other databases.

Databases	Search Strategy
SCOPUS	TITLE-ABS-KEY(({Patient Safety} OR {Patient Safeties} OR {Safeties, Patient} OR {Safety, Patient}) AND (Students OR Student OR {School Enrollment} OR {Enrollment, School} OR {Enrollments, School} OR {School Enrollments} OR Learners OR Scholars OR Undergraduates OR Graduates OR Academics OR Interns OR Apprentices) AND (Instrumentation OR Instruments OR Implements OR Devices OR Apparatus OR Equipment OR Utensils OR Gadgets OR Appliances OR Contrivances OR Mechanisms OR Machines OR Apparatuses OR Gear OR Hardware OR Resources))
EMBASE	(‘Patient Safety’:ab,ti OR ‘Patient Safeties’:ab,ti OR ‘Safeties, Patient’:ab,ti OR ‘Safety, Patient’:ab,ti) AND (Students:ab,ti OR Student:ab,ti OR ‘School Enrollment’:ab,ti OR ‘Enrollment, School’:ab,ti OR ‘Enrollments, School’:ab,ti OR ‘School Enrollments’:ab,ti OR Learners:ab,ti OR Scholars:ab,ti OR Undergraduates:ab,ti OR Graduates:ab,ti OR Academics:ab,ti OR Interns:ab,ti OR Apprentices:ab,ti) AND (Instrumentation:ab,ti OR Instruments:ab,ti OR Implements:ab,ti OR Devices:ab,ti OR Apparatus:ab,ti OR Equipment:ab,ti OR Utensils:ab,ti OR Gadgets:ab,ti OR Appliances:ab,ti OR Contrivances:ab,ti OR Mechanisms:ab,ti OR Machines:ab,ti OR Apparatuses:ab,ti OR Gear:ab,ti OR Hardware:ab,ti OR Resources:ab,ti)
